# Factor Structure and Gender Invariance of Chinese Version State-Trait Anxiety Inventory (Form Y) in University Students

**DOI:** 10.3389/fpsyg.2020.02228

**Published:** 2020-10-08

**Authors:** Yan Han, Jie Fan, Xiang Wang, Jie Xia, Xingze Liu, Huan Zhou, Yi Zhang, Xiongzhao Zhu

**Affiliations:** ^1^Medical Psychological Center, The Second Xiangya Hospital, Central South University, Changsha, China; ^2^Medical Psychological Institute, Central South University, Changsha, China; ^3^National Clinical Research Center for Mental Disorders, Changsha, China

**Keywords:** state anxiety, trait anxiety, State-trait anxiety inventory, factor structure, Gender invariance

## Abstract

**Background:**

Anxiety can be classified as state anxiety and trait anxiety which present the current level of anxiety and the generalized anxiety tendencies of individuals, respectively. The State-Trait Anxiety Inventory form Y (STAI-Y) is a reliable instrument used to test both the levels of state and trait anxiety across various countries. However, the optimal factor structure of STAI-Y in different populations is not consistent and is not clear in Chinese university students. In addition, the gender invariance is the premise for comparing the scores of STAI-Y between men and women which also need to be verified. Therefore, this study explored the optimal factor structure of STAI-Y and examined whether the optimal factor structure satisfied measurement invariance across gender in Chinese university students.

**Method:**

A sample of 2117 Chinese university students participated in this study including 748 men and 1369 women. The optimal factor structure was decided by singer group confirmatory factor analysis (CFA) and exploratory structural equation modeling (ESEM). Furthermore, the configural invariance, metric invariance, scalar invariance, and strict invariance models were administrated in multigroup CFA to detect the measurement equivalence of STAI-Y across gender in Chinese university students. The reliability of STAI-Y was tested by Cronbach’s alpha coefficient and McDonald’s omega coefficients.

**Results:**

The optimal factor structure of STAI-Y was four-factor model and reached strict gender invariance in Chinese university students. Moreover, the STAI-Y also had adequate reliability in Chinese university students.

**Conclusion:**

This study explored the factor structure and gender invariance of STAI-Y in Chinese university students. In sum, the four-factor structure of STAI-Y obtained the best goodness-of-fit and satisfied gender invariance which deepened the understanding of STAI-Y in Chinese university students.

## Introduction

Anxiety is an emotional status including nervous, apprehensive and tensed feelings (Vitasari et al., 2011), which are common in the lives of healthy people and have become clinical symptoms of anxiety disorders, depression as well as other mental illnesses (Kabacoff et al., 1997; Bui and Fava, 2017). Anxiety has complex constructs on the basis of different theories, Spielberger (1966) suggested that anxiety should be separated into trait anxiety and state anxiety. Specifically, trait anxiety refers to individual’s relatively stable response to threat and stress, is usually related to personality, and reflects individual differences, while state anxiety is defined as emotional arousal and unpleasant feelings to the threatening situation which can change along with time and circumstances (Shedletsky and Endler, 1974; Andrade et al., 2001; Endler and Kocovski, 2001). Whereafter, the classification of trait anxiety and state anxiety has been recognized and applied by many scholars in the field of psychology (Endler and Kocovski, 2001).

In order to test the levels of state anxiety and trait anxiety, Spielberger et al. (1983) developed the original State-Trait Anxiety Inventory and revised it as State-Trait Anxiety Inventory form Y (STAI-Y) in 1983 which included 20 items for state anxiety and 20 items for trait anxiety (Shek, 1988). The STAI-Y showed satisfactory reliability and validity in numerous countries such as Greece (Fountoulakis et al., 2006), France (Bouchard et al., 1998), Malaysia (Vitasari et al., 2011), the United States (Kabacoff et al., 1997), Portugal (Andrade et al., 2001), Lebanon (Hallit et al., 2019), Caribbean (Maynard et al., 2010), and so on. These studies included general population and psychiatric patients. In terms of the application of STAI-Y in China, we found several studies. For example, Cao and Liu (2015a) test the factor structure and measurement invariance of STAI-Y in Chinese children and adolescents; Zhang and Gao (2012) explored the validation of the Trait Anxiety Scale for STAI-Y in Suicide Victims and Living Controls of Chinese Rural Youths; Ma et al. (2013) evaluated the psychometric properties of STAI-Y in Taiwanese outpatients with anxiety disorders. Although STAI-Y has been developed for many years and has been used in China for a long time, there are still many controversial questions to be addressed.

For the factor structure of STAI-Y, we found that it varies among foreign studies. Some researchers found there was a four-factor structure, that is state-present anxiety, state-absent anxiety, trait-present anxiety, and trait-absent anxiety, which revealed both of the theoretical and methodological bases of STAI-Y including the state-trait anxiety distinction and the anxiety-present/absent distinction (Spielberger et al., 1983; Gauthier and Bouchard, 1993; Suzuki et al., 2000). Particularly, the four-factor solutions was recognized by many previous scholars (Spielberger et al., 1980; van der Ploeg et al., 1980; Bernstein and Eveland, 1982). The typical two-factor structure of STAI-Y consisting of state anxiety and trait anxiety or consisting of anxiety-present and anxiety-absent reported by most studies (Kabacoff et al., 1997; Iwata and Mishima, 1999; Maynard et al., 2010; Vitasari et al., 2011). It is worth noting that anxiety-absent/present two factors distinction was more popular than state-trait anxiety two factors distinction in many researches, especially in clinical patients, and the Japanese general population (Iwata and Mishima, 1999). In addition, there are other factor models for STAI-Y. For example, Hallit et al. (2019) found three factors for state anxiety and four factors for trait anxiety in Lebanese population; Vigneau and Cormier (2008) found that the two-construct, two-method model is superior to the one-factor, two-factor, and four-factor models in French-Canadian university students and French adults; The principal components analysis revealed seven factors of STAI-Y in Greek healthy volunteers and depressed patients (Fountoulakis et al., 2006). In Chinese studies, the factor structure was also incongruent. Cao and Liu (2015a) compared 11 factor models indicating that the state-trait anxiety factor structure obtained the best goodness-of-fit indices in Chinese children and adolescents. Similarly, Chen et al. (2013) found a state-trait anxiety two factor structure in Chinese migrant children. While Ma et al. (2013) found a four-factor structure in Taiwanese outpatients with anxiety disorders. Besides, Li and Qian (1995) revised the norm of STAI-Y in Chinese university students which also illustrated that the four-factor structure is the most appropriate factor model. However, the sample size is small and the confirmatory factor analysis (CFA) was not conducted in the Li and Qian’s study.

Furthermore, the gender differences of the scores on STAI-Y were inconsistent in previous studies. Part researches revealed that the performance on STAI-Y between men and women had significant differences (Andrade et al., 2001; Zhang et al., 2005; Jia et al., 2016), while other part studies demonstrated that men had similar STAI-Y scores with women (Li and Qian, 1995; Li et al., 2006; Cooper et al., 2007; Ma et al., 2013). Actually, the gender invariance of STAI-Y is the premise of comparing the scores of STAI-Y between men and women (Zhou et al., 2019).

From the above, the STAI-Y has been widely used as a reliable instrument to measure the level of anxiety in China (Zhang et al., 2005; Zhang and Gao, 2012; Ma et al., 2013; Cao and Liu, 2015a, b). However, as one of the important characteristics, the factor structure is unclear in Chinese college students. Further, many studies have compared the scores of STAI-Y between men and women (Li and Qian, 1995; Zhang et al., 2005; Li et al., 2006; Ma et al., 2013), but no study proved gender invariance of STAI-Y in Chinese university students. Therefore, one aim of our study is to explore the optimal factor structure of STAI-Y in a large sample of Chinese college students and another is to explore whether the best fit factor model of STAI-Y meets the measurement invariance across gender in our sample.

## Materials and Methods

### Participants

This survey was administrated in Hunan University of Chinese Medicine between October and November, 2019. A total of 2278 new college students were invited to participate in our study, and we collected 2278 questionnaires overall. After our screening, we found 154 invalid questionnaires including various incomplete data and multivariate outliers, moreover, 7 participants had history of psychiatric disorders. We removed these undesirable questionnaires and finally there were 2117 undergraduates included in this study, all of whom voluntarily completed the written informed consent. There were 748 men and 1369 women. The age of men ranged from 16 to 24 years (*M* = 19.26; SD = 0.81) and the age of women ranged from 16 to 23 years (*M* = 19.14; SD = 0.71).

### Instruments

The STAI-Y (Spielberger et al., 1983) is a self-report instrument testing state anxiety and trait anxiety. State anxiety is a temporary emotional state which requires individuals to mainly consider the present feelings. In contrast, trait anxiety is a relatively stable response to stressful situations which requires individuals to consider the general feelings of anxiety that they experience all the time. The Chinese version STAI-Y contained 40 items, half of them assess state anxiety and the other half measure trait anxiety. All items rated from 1 (not at all) to 4 (very much so). In addition, there are 19 items (1, 2, 5, 8, 10, 11, 15, 16, 19, 20, 21, 23, 26, 27, 30, 33, 34, 36, and 39) performing absent anxiety which need to be reversely scored. The total score of STAI-Y was accumulated by the sum of all items. Higher scores indicated higher anxiety level.

### Statistical Analysis

We used the softwares of SPSS version 25.0 and Mplus version 7.0 in this study. Single group CFAs were administrated to obtain the fit indices of the following factor models: one-factor model, state-trait anxiety factor model, anxiety-absent/present factor model, four-factor model (state anxiety absent; state anxiety present; trait anxiety absent; and trait anxiety present), and correlated trait – correlated method minus one model. It’s worth noting that the correlated trait – correlated method minus one model was used to examine the method effect in STAI-Y. Moreover, the exploratory structural equation modeling (ESEM) was implemented to verify the optimal factor structure among one factor model, two factor model and four factor model. Given that the analysis data were categorical variables, the weighted least squares means and variance adjusted (WLSMV) estimator was employed in all CFAs and ESEM. Due to the fact that the value of chi-square (χ^2^) is sensitive to sample size, according to previous research, the comparative fit index (CFI), Tucker-Lewis index (TLI), and root-mean-square error of approximation (RMSEA) were sufficient to assess the goodness-of-fit. Generally, CFI and TLI greater than 0.90, and RMSEA less than 0.08 indicate that the model fit is acceptable (Hu and Bentler, 1999; Chaix et al., 2017). The fitting indicators of the optimal model should be the best (Sun, 2005). Furthermore, we conducted multigroup CFAs to test the gender invariance of the optimal model of STAI-Y. There were four stepwise processes testing configural invariance, metric invariance, scalar invariance and strict invariance, which can examine whether the composition of factor structure, factor loadings of variables, intercepts of each item and latent variable variation are equal across gender, respectively, (Han et al., 2020). Similarly, the sample size can easily disturb the χ^2^ change test, therefore, the equivalent model is considered acceptable when the value of the difference of CFI (ΔCFI) was less than 0.010 (Li et al., 2019). Because the Kolmogorov–Smirnov normality test showed that the data was not satisfied by the normal distribution, the mean differences of STAI-Y between men and women were tested by Mann–Whitney *U* tests. The reliability parameters were evaluated by Cronbach’s alpha coefficients and McDonald’s omega coefficients. Furthermore, we tested the correlations of latent variables of STAI using a multiple-indicator multiple-cause (MIMIC) approach.

## Results

### Descriptive Statistics

The average score for each item is 1.227 at the lowest and 3.148 at the highest. All items had significant *P* values of skewness and kurtosis which were summarized in Table 1.

**TABLE 1:** Descriptive data of STAI-Y in total sample.

	*M*	SD	Skewness	Kurtosis	Standardized factor loadings
**State anxiety absent**					
Item1	2.890	0.807	–0.205	–0.649	0.691
Item2	3.148	0.800	–0.622	–0.258	0.734
Item5	2.558	0.897	–0.026	–0.765	0.684
Item8	2.464	0.899	–0.061	–0.781	0.826
Item10	2.668	0.863	–0.124	–0.660	0.855
Item11	2.696	0.858	–0.095	–0.693	0.754
Item15	2.667	0.898	–0.103	–0.786	0.842
Item16	2.552	0.911	–0.067	–0.798	0.861
Item19	2.613	0.844	–0.022	–0.629	0.787
Item20	2.751	0.854	–0.157	–0.671	0.864
**State anxiety present**					
Item3	1.664	0.770	0.942	0.220	0.713
Item4	1.512	0.741	1.364	1.223	0.773
Item6	1.815	0.801	0.727	–0.055	0.774
Item7	1.405	0.700	1.795	2.801	0.777
Item9	1.414	0.659	1.550	1.972	0.746
Item12	1.466	0.760	1.613	1.903	0.779
Item13	1.227	0.563	2.789	8.048	0.857
Item14	1.973	0.901	0.675	–0.307	0.522
Item17	1.745	0.753	0.783	0.193	0.825
Item18	1.496	0.691	1.273	1.095	0.822
**Trait anxiety absent**					
Item21	2.890	0.749	–0.201	–0.402	0.822
Item23	2.499	0.850	0.042	–0.614	0.756
Item26	2.576	0.827	0.060	–0.587	0.760
Item27	2.550	0.847	0.078	–0.634	0.761
Item30	2.799	0.788	–0.144	–0.517	0.876
Item33	2.880	0.828	–0.228	–0.669	0.819
Item34	2.372	0.871	0.161	–0.646	0.536
Item36	2.629	0.853	–0.061	–0.645	0.868
Item39	2.594	0.801	0.055	–0.524	0.728
**Trait anxiety present**					
Item22	1.534	0.717	1.237	1.051	0.790
Item24	2.462	1.028	–0.005	–1.143	0.300
Item25	1.422	0.705	1.709	2.475	0.788
Item28	1.778	0.815	0.837	0.084	0.697
Item29	1.898	0.860	0.746	–0.084	0.665
Item31	1.717	0.803	0.917	0.176	0.755
Item32	1.989	0.886	0.689	–0.184	0.671
Item35	1.691	0.744	0.913	0.503	0.735
Item37	1.938	0.881	0.620	–0.426	0.720
Item38	1.552	0.793	1.365	1.150	0.826
Item40	2.030	0.865	0.485	–0.480	0.607

*Note: All of the standardized factor loadings are statistically significant.*

### Confirmatory Factor Analysis and Exploratory Structural Equation Modeling

In CFAs, all fit indices of the one-factor model and state-trait anxiety factor model did not reach the required standard in total sample and gender groups. For the anxiety-absent/present factor model and four-factor model (state anxiety absent; state anxiety present; trait anxiety absent; and trait anxiety present), both the values of CFA and TLI were higher than 0.90 and the value of RMSEA was lower than 0.08. Specifically, the fit indices of anxiety-absent/present factor model were as followings: χ^2^ = 9119.634, df = 739, CFI = 0.919, TLI = 0.914, and RMSEA = 0.073 (total sample); χ^2^ = 3431.141, df = 739, CFI = 0.926, TLI = 0.922, and RMSEA = 0.070 (men); and χ^2^ = 5863.689, df = 739, CFI = 0.924, TLI = 0.920, and RMSEA = 0.071 (women). Comparatively speaking, the four-factor model (state anxiety absent; state anxiety present; trait anxiety absent; and trait anxiety present) had a better goodness-of-fit than anxiety-absent/present factor model and was the best fit model with the following fit indices: χ^2^ = 8019.482, df = 734, CFI = 0.929, TLI = 0.925, and RMSEA = 0.068 (total sample); χ^2^ = 3108.040, df = 734, CFI = 0.935, TLI = 0.931, and RMSEA = 0.066 (men); χ^2^ = 5130.041, df = 734, CFI = 0.935, TLI = 0.931, and RMSEA = 0.066 (women). Moreover, the correlated trait-correlated method minus one model also reached all fitting requirements and performed a similar goodness-of-fit to the anxiety-absent/present factor model. The standardized factor loadings of the four-factor model (state anxiety absent; state anxiety present; trait anxiety absent; and trait anxiety present) in CFA ranged from 0.300 to 0.876 which are exhibited in Table 1. Other specific information is shown in Table 2. In ESEM, neither one-factor structure nor two factor structure can meet the fitting requirements totally, but the four factor structure reached a satisfactory goodness-of-fit which is exhibited in Table 3.

**TABLE 2:** Goodness-of-fit indices of CFA in demographic subgroups.

Item		χ^2^	df	CFI	TLI	RMSEA (90%CI)
Model 1	Total	26546.192	740	0.750	0.736	0.128(0.127 0.130)
	Men	9346.587	740	0.765	0.752	0.125(0.122 0.127)
	Women	16430.411	740	0.769	0.756	0.124(0.123 0.126)
Model 2	Total	25300.119	739	0.762	0.749	0.125(0.124 0.127)
	Men	8970.266	739	0.775	0.762	0.122(0.120 0.124)
	Women	15661.948	739	0.780	0.768	0.121(0.120 0.123)
Model 3	Total	9119.634	739	0.919	0.914	0.073(0.072 0.075)
	Men	3431.141	739	0.926	0.922	0.070(0.067 0.072)
	Women	5863.689	739	0.924	0.920	0.071(0.069 0.073)
Model 4	Total	8019.482	734	0.929	0.925	0.068(0.067 0.070)
	Men	3108.040	734	0.935	0.931	0.066(0.063 0.068)
	Women	5130.041	734	0.935	0.931	0.066(0.064 0.068)
Model 5	Total	8764.712	718	0.922	0.915	0.073(0.071 0.074)
	Men	3271.151	718	0.930	0.924	0.069(0.067 0.071)
	Women	5685.153	718	0.927	0.920	0.071(0.069 0.073)

*Note: Model 1 = one factor; Model 2 = state anxiety + trait anxiety; Model 3 = anxiety absent + anxiety present; Model 4 = state anxiety absent + state anxiety present + trait anxiety absent + trait anxiety present; Model 5 = Correlated Trait-Correlated Method minus one model; CFI, comparative fit index; TLI, Tucker-Lewis index; and RMSEA, root-mean-square error of approximation.*

**TABLE 3:** Goodness-of-fit indices of ESEM in demographic subgroups.

Item		χ^2^	df	CFI	TLI	RMSEA (90%CI)
One factor model	Total	26546.192	740	0.750	0.736	0.128(0.093 0.096)
	Men	9346.586	740	0.765	0.752	0.125(0.122 0.127)
	Women	16430.411	740	0.769	0.756	0.124(0.123 0.126)
Two factor model	Total	11789.542	701	0.893	0.880	0.086(0.085 0.088)
	Men	3989.130	701	0.910	0.900	0.079(0.077 0.082)
	Women	7417.148	701	0.901	0.890	0.084(0.082 0.085)
Four factor model	Total	5804.862	626	0.950	0.937	0.063(0.061 0.064)
	Men	2106.046	626	0.960	0.950	0.056(0.054 0.059)
	Women	3795.770	626	0.953	0.942	0.061(0.059 0.063)

*Note: CFI, comparative fit index; TLI, Tucker-Lewis index; and RMSEA, root-mean-square error of approximation.*

### Gender Invariance of STAI-Y Four-Factor Model

According to the results of single CFAs and ESEM, the four-factor structure (state anxiety absent; state anxiety present; trait anxiety absent; and trait anxiety present) was used as the baseline model in the measurement invariance testing. The fit indices of the configural invariance model (χ^2^ = 8094.188, df = 1468, CFI = 0.936, TLI = 0.932, and RMSEA = 0.065) were all acceptable. In addition, both the values of ΔCFI between nested models were 0.001 which indicated that the four-factor structure reached strict invariance between men and women. The specific information is summarized in Table 4.

**TABLE 4:** Gender invariance testing of the SATI-Y.

Model	χ^2^	df	CFI	TLI	RMSEA (90%CI)	ΔCFI
Configural invariance	8094.188	1468	0.936	0.932	0.065(0.064 0.067)	
Metric invariance	8029.837	1536	0.937	0.936	0.063(0.062 0.065)	0.001
Scalar invariance	8025.704	1580	0.938	0.938	0.062(0.061 0.063)	0.001

*Note: CFI, comparative fit index; TLI, Tucker-Lewis index; RMSEA, root-mean-square error of approximation; and ΔCFI, change in comparative fit index.*

### Reliability and Correlations Among the Latent Variables of STAI-Y

The Cronbach’s alpha coefficients of four factors ranged from 0.868 to 0.921 in total sample; ranged from 0.875 to 0.920 in the men sample; and ranged from 0.865 to 0.921 in the women sample. The McDonald’s omega coefficients of four factors ranged from 0.691 to 0.800 in total sample; ranged from 0.706 to 0.800 in the men sample; and ranged from 0.684 to 0.801 in the women sample. The specific results including 90% CI are displayed in Table 5.

**TABLE 5:** The reliability parameters of STAI-Y in this study.

		Cronbach’s alpha coefficients (90%CI)	McDonald’s omega coefficient (90%CI)
Total (*N* = 2118)	State anxiety absent	0.921(0.916 0.925)	0.800 (0.790 0.810)
	State anxiety present	0.874(0.867 0.881)	0.733(0.717 0.748)
	Trait anxiety absent	0.896(0.890 0.901)	0.775(0.764 0.786)
	Trait anxiety present	0.868(0.861 0.875)	0.691(0.678 0.705)
Men (*N* = 748)	State anxiety absent	0.920(0.912 0.927)	0.800(0.790 0.810)
	State anxiety present	0.876(0.864 0.887)	0.726(0.699 0.753)
	Trait anxiety absent	0.899(0.890 0.908)	0.782(0.763 0.800)
	Trait anxiety present	0.875(0.863 0.885)	0.706(0.683 0.728)
Women (*N* = 1370)	State anxiety absent	0.921(0.916 0.926)	0.801(0.789 0.813)
	State anxiety present	0.873(0.864 0.881)	0.724(0.705 0.744)
	Trait anxiety absent	0.894(0.887 0.901)	0.773(0.760 0.787)
	Trait anxiety present	0.865(0.856 0.874)	0.684(0.667 0.701)

Controlling the effects of age, all correlations among the latent variables of STAI-Y were significant with *P* < 0.01. Specifically, the correlations were moderate and negative between anxiety absent factors and anxiety present factors (absolute value: 0.485–0.546, total; 0.485–0.537, men; and 0.489–0.557, women) but high and positive between state anxiety absent and trait anxiety absent (0.893, total; 0.888, men; and 0.898, women) as well as between state anxiety present and trait anxiety present (0.838, total; 0.853, men; and 0.828, women; Table 6)

**TABLE 6:** Correlations among the latent variables of STAI-Y using MIMIC approach.

	State anxiety absent	State anxiety present	Trait anxiety absent
**Total sample**			
State anxiety absent	1		
State anxiety present	−0.546**	1	
Trait anxiety absent	0.893**	−0.485**	1
Trait anxiety present	−0.528**	0.838**	−0.526**
**Men**			
State anxiety absent	1		
State anxiety present	−0.537**	1	
Trait anxiety absent	0.888**	−0.485**	1
Trait anxiety present	−0.518**	0.853**	−0.536**
**Women**			
State anxiety absent	1		
State anxiety present	−0.557**	1	
Trait anxiety absent	0.898**	−0.489**	1
Trait anxiety present	−0.535**	0.828**	−0.520**

Note:**P < 0.01; MIMIC, multiple-indicator multiple-cause; and The effects of age was controlled in the analysis.

### Mann–Whitney *U* Tests

As presented in Table 7, the significant differences across gender were just found in the state anxiety absent dimension and trait anxiety absent dimension (*P* < 0.05). However, both effect sizes were lower than 0.3 (Fritz et al., 2012).

**TABLE 7:** Comparison of STAI-Y scores between different gender.

Variables		*M*	SD	Mean difference	*P*	*r*
State anxiety absent	Men	27.487	6.764			
	Women	26.743	6.491	0.744	0.010	0.056
State anxiety present	Men	15.971	5.237			
	Women	15.575	4.952	0.396	0.162	
Trait anxiety absent	Men	24.102	5.689			
	Women	23.607	5.371	0.495	0.031	0.045
Trait anxiety present	Men	19.944	6.186			
	Women	20.038	5.912	0.094	0.435	
STAI	Men	76.642	6.054			
	Women	76.403	5.721	0.239	0.114	

## Discussion

This study explored the optimal factor structure of the Chinese version of STAI-Y and whether the best fit factor model reached measurement invariance across gender in university students.

According to previous studies, the factor structure varied in different populations and cultures (Bernstein and Eveland, 1982; Vigneau and Cormier, 2008; Vitasari et al., 2011; Hallit et al., 2019). In Chinese research, the factor structure was also not consistent (Chen et al., 2013; Ma et al., 2013; Cao and Liu, 2015a). Therefore, it is important to figure out what the best factor structure of STAI-Y is in Chinese university students. Actually, the typical factor structures included state-trait anxiety factors, anxiety absent/present factors and four-factors (state anxiety absent, state anxiety present, trait anxiety absent, and trait anxiety present). Therefore, we administrated CFA for these factor models as well as a one-factor model which deemed that all 40 items loaded onto one factor measured anxiety level. Finally, we found that the four-factor model (state anxiety absent, state anxiety present, trait anxiety absent, and trait anxiety present) was superior to other factor models in total sample and subgroups with satisfactory goodness-of-fit. Besides, the results of ESEM also verified that the four-factor model (state anxiety absent, state anxiety present, trait anxiety absent, and trait anxiety present) was the best fit factor structure. Actually, the ESEM not only exhibited the advantages of exploratory factor analysis and CFA, but also showed new advantages in research and application (Asparouhov and Muthén, 2009; Marsh et al., 2009). For example, the results concluded by ESEM were closer to the real situation (Marsh et al., 2011). Therefore, in this study, ESEM and CFA were combined to determine the optimal factor structure of STAI-Y, which could obtain more objective and realistic results than previous studies. Moreover, the correlated trait-correlated method minus one model did not improve the fit to the data over the anxiety-absent/present factor model which claimed that the negative-item substantively separated from positive-item regarding as a meaningful factor construct (Forsterlee and Ho, 1999). In terms of the standardized factor loadings, the lowest value was 0.300 for item 24 “I wish I could be as happy as others seem to be” which also be found in previous Chinese research (Li and Qian, 1995) and according to Li and Qian (1995), the reasonable interpretations may be that the “unhappy” was classified in depression checklist rather than in anxiety checklist by Chinese psychologists and the rhetorical problems about the Chinese translation of item 24. Due to the standardized factor loading of item 24 was not lower than 0.300, we didn’t remove it in this study (Cordova et al., 2019).

In order to examine whether the four-factor structure (state anxiety absent, state anxiety present, trait anxiety absent, and trait anxiety present) reached a measurement invariance across gender groups, we tested configural invariance model, metric invariance model, scalar invariance model, and strict invariance model in multi-group CFA successively. Finally, we confirmed that the four-factor structure (state anxiety absent, state anxiety present, trait anxiety absent, and trait anxiety present) achieved strict gender invariance in Chinese university students and the Mann–Whitney *U* tests revealed significant differences in state anxiety absent dimension and trait anxiety absent dimension between men and women.

Consistently with previous researches, both Cronbach’s alpha coefficients and McDonald’s omega coefficients showed adequate reliability of four factor dimensions (state anxiety absent, state anxiety present, trait anxiety absent, and trait anxiety present) in total sample and subgroups. In addition, four factors of STAI-Y were significantly correlated with each other when controlling the effect of age and the anxiety-absent factors were negatively related to anxiety-present factors which conformed to the theoretical and methodological basis of STAI-Y.

One limitation in our study was the unitary sample which was just taken from one university in the Hunan Province of China. Moreover, we had no data to discuss the test-retest reliability parameter and more types of validity about STAI-Y in Chinese college students. Therefore, related contents need to be supplemented and explored by further studies.

In summary, our study confirmed that the STAI-Y had a four-factor structure in Chinese university students which reached strict invariance across gender. Moreover, the STAI-Y was also a reliable instrument in Chinese university students.

This study was approved by the Ethics Committee of the Second Xiangya Hospital, Central South University.

## Data Availability Statement

The datasets generated for this study are available on request to the corresponding author.

## Ethics Statement

The studies involving human participants were reviewed and approved by The Ethics Committee of the Second Xiangya Hospital of Central South University. The participants provided their written informed consent to participate in the study.

## Author Contributions

YH and XZ conceived and designed the study. YH, JF, and XW collected the data and revised the manuscript. YH, JX, and XL organized and supervised data collection and inputting. YH drafted the manuscript, organized and supervised the data analysis. HZ and YZ provided critical comments on various drafts of the manuscript. All authors read and approved the final manuscript.

## Conflict of Interest

The authors declare that the research was conducted in the absence of any commercial or financial relationships that could be construed as a potential conflict of interest.
